# Editorial: Insights into non-coding RNAs in programmed cell death, from molecular mechanism of tumorigenesis to therapeutic targets

**DOI:** 10.3389/fonc.2023.1239782

**Published:** 2023-06-27

**Authors:** Jinhui Liu, Peixin Dong

**Affiliations:** ^1^ Department of Gynecology, First Affiliated Hospital, Nanjing Medical University, Nanjing, China; ^2^ Department of Obstetrics and Gynecology, Hokkaido University School of Medicine, Hokkaido University, Sapporo, Japan

**Keywords:** non-coding RNA, microRNA, long non-coding RNA, circular RNA, cell death, cancer biomarker, cancer therapeutic resistance

Cancer treatment has been hindered by cells that resist cell death, but inducing programmed cell death (PCD) shows promise as a strategy to control tumor growth. A large portion of transcripts is non-coding RNAs (ncRNAs), many of which have been implicated in cancer development, spread, and resistance, as well as in the regulation of programmed cell death according to the human transcriptome. To understand the root cause of cancer and develop customized therapies, in this Research Topic, we addressed the interplay between ncRNAs and PCD regulation, and the impact of ncRNAs associated with programmed cell death on tumor progression.

Qin et al. investigate the role of the F-actin capping protein a1 subunit (CAPZA1) in lung adenocarcinoma (LUAD) and its potential as a therapeutic target. The study found that CAPZA1 is overexpressed in multiple cancers, including LUAD, and is associated with worse survival in several cancers. The study also identified a potential competitive endogenous RNA network involving CAPZA1, miR-30d-5p, and AC026356.1 in LUAD. Additionally, the study found that CAPZA1 was positively correlated with immune cell infiltration and immune checkpoint expression, suggesting its potential as a target for immunotherapy. This paper discusses various studies related to cancer research, including immunogenomic analyses, tumor mutational burden, microsatellite instability, noncoding RNA therapeutics, and gene set enrichment analysis. It also highlights the importance of understanding the genotype-immunophenotype relationship and predictors of response to checkpoint blockade in cancer treatment. The article further explores the role of microRNAs and ceRNA crosstalk in cancer progression and the potential of immune therapy with chemotherapy in the era of immune checkpoint inhibitors.

Zhou et al. discover that the three predictive autophagy-related lncRNAs (SMURF2P1, MIR9-3HG, and AC005332.4), all of which are independent prognostic indicators in cervical cancer. Following that, they calculate the risk ratings for the three lncRNAs and create a signature of autophagy-relevant lncRNAs. Patients were split into two categories, and those with greater risk ratings had worse survival. Their findings indicate a link between these autophagy-related lncRNAs and tumor-immune infiltration. Finally, the authors discovered that SMURF2P1 was upregulated, while MIR9-3HG and AC005332.4 are downregulated in cervical cancer cells. This signature might provide effective and valuable clinical applications for accurate prognostic prediction and individualized treatment of cervical cancer patients.

Guo et al. utilized RNA-seq data from normal and breast cancer (BC) tissues to identify differentially upregulated lncRNAs, cuproptosis-related lncRNAs, and predictive lncRNAs. They built the risk model using LASSO regression analysis on nine cuproptosis-associated lncRNAs. The researchers discovered nine predictive lncRNAs linked to cuproptosis in order to build a risk model for BC patients. BC patients in the low-risk category had a lower total mortality rate. Cellular biochemical pathways and immune cell stimulation were more prevalent in the high-risk categories. In the high-risk groups, CD8^+^ T cells were downregulated, whereas *PD-1* mRNA was increased in the low-risk groups. BC patients who had reduced cuproptosis-related lncRNA risk ratings had greater rates of CD8^+^ T-cell invasion, which resulted in improved immunotherapy outcomes. This research indicates that targeting cuproptosis-associated lncRNAs could be a game changer in cancer treatment in the future.

Zhang et al. construct a model based on a cuproptosis-related lncRNA signature and further evaluate the function of lncRNA LINC00592 using lung cancer cell lines. Following the knockdown of LINC00592, there was also a significant reduction in cell proliferation and migration, suggesting that this cuproptosis-related lncRNA signature is capable of anticipating the prognosis of lung cancer patients and may provide direction for lung cancer treatment.

Qian et al. investigated prior research on miR-1258 in human tumors. MiR-1258 expression is commonly downregulated in tumor cells. MiR-1258 has been shown to act as a tumor suppressor in human tumors by blocking metastasis, stemness, EMT, and glycolysis while increasing cell death and chemosensitivity. Furthermore, miR-1258 can be employed as an early cancer diagnosis and prognostic predictor. As a result, miR-1258 may open up new avenues for precision medicine by acting as a new biomarker as well as a potential target for tumor therapy.

Peng et al. address the mechanisms that underlie EGFR-TKI resistance and its therapeutic consequences in non-small cell lung cancer. (NSCLC). They discuss existing EGFR-TKI-based combination therapy as well as new treatment strategies for assaulting EGFR-TKI resistance in NSCLC, such as nanoparticles containing non-coding RNAs. The authors determine that EGFR-TKI combination treatments are successful therapy options for NSCLC; however, more studies are needed to overcome EGFR-TKI resistance.

According to the articles in this Research Topic, ncRNAs play an essential part in PCD regulation and, as a consequence, impact cancer development and treatment resistance. Further research into the molecular processes of PCD-related non-coding RNAs could deliver fresh knowledge into the formation of human cancers and be helpful in creating innovative personalized anticancer therapies.

## Author contributions

All authors listed have made a substantial, direct, and intellectual contribution to the work and approved it for publication.

